# Experimental Investigation of a Pilot Solar-Assisted Permeate Gap Membrane Distillation

**DOI:** 10.3390/membranes11050336

**Published:** 2021-04-30

**Authors:** Mohammed M. Alquraish, Sami Mejbri, Khaled A. Abuhasel, Khalifa Zhani

**Affiliations:** 1 Department of Mechanical, College of Engineering, University of Bisha, P.O. Box 199, Bisha 61922, Saudi Arabia; malqraish@ub.edu.sa (M.M.A.); kabuhasel@ub.edu.sa (K.A.A.); 2 Laboratory of Electromechanical Systems (LASEM), National Engineering School of Sfax, University of Sfax, Sfax 3038, Tunisia; sami.mejbri@stud.enis.tn

**Keywords:** distillate quality, solar desalination, PGMD, membrane distillation, solar water collectors, photovoltaic solar collectors

## Abstract

This research deals with the process of water desalination, involving an experimental design used to study a new prototype of a solar membrane distillation plant based on the weather conditions of Kairouan City, Tunisia. In this experiment, the pilot is left autonomous with the sun as the only source of energy. The operating process of a desalination plant consists of solar energy provided by the sun using solar energy collectors, which provide energy through their photovoltaic panels for heating brackish water. Additionally, the membrane used in this study was of the spiral wound design, which allowed for a compact arrangement besides effective internal heat recovery. The system start-up was successfully carried out and experimental studies were launched on various days of August 2020. During the experiment, the average production was approximately 15.92 L/m^2^ ap per day while the distillate’s electoral conductivity amounted to 1865 μS/cm. Calculations revealed that the specific thermal energy consumption for the system ranged between 90 and 310 kWh/m^3^.

## 1. Introduction

Water scarcity is a growing issue for a rising global population which is expected to surpass 8.3 billion people by 2030 [1]. In a 2013 study [2], it was estimated that approximately 700 million people were suffering from the scarcity of water and this number is expected to go up to 2.8 billion by the year 2025. On the other hand, the global demand for freshwater is predicted to rise by over 50% by 2050 [1]. For this reason, seawater desalination is widely regarded as a solution to the problem of dwindling freshwater resources, especially in arid and semi-arid regions. In 2000, operational desalination plants in various parts of the world generated an estimated 7 million m^3^/day [3] of freshwater, while today, this capacity has increased to reach 78.4 million m^3^/day [4]. However, about 8–17% of these desalination plants are using thermally powered MED (multi-effect distillation) and MSF (multi-stage flash) technology versus 70% that are using electrically driven reverse osmosis (RO) technology, which makes them very high capacity desalination but capital and energy intensive plants [3]. However, only 3.5% of the desalination capacity of the world is generated by small-scale plants (less than 1000 m^3^/day) [3], which is needed mainly in rural areas where 80% of the population has no access to fresh drinking water. Moreover, the three desalination technologies mentioned above require a significant amount of chemicals for smooth operation, which creates an additional cost and logistical hassle in rural areas. As such, there is rising demand for the enhancement of small-scale, chemical-free, and operationally non-complex desalination technology, preferably coupled with a renewable energy system that can be implemented in rural areas. On the other hand, membrane distillation (MD), as a desalination method, can largely meet the above criteria since its scalability makes it suitable for a wide range of applications vis-à-vis the operational capacity Although the distillation process itself does not rely on any chemical treatment, as it is determined by properties of the inlet feed water, some kind of pre-treatment might be necessary for a longer operational life. Besides, the easy coupling with renewable energy (notably solar thermal energy) makes MD especially suitable for stand-alone and off-grid applications [5,6].

## 2. Solar-Assisted Membrane Distillation Systems—State of the Art

Some of the many exploration endeavors to utilize sun-based energy to supply the MD with power are presented in the accompanying passages. For example, M. Burhan et al. [7] tried the vacuum membrane distillation (VMD) arrangement framework from MEMSYS. This framework involves 4 VMD portions in an arrangement that is made out of absolute four VMD modules that are working in single and multi-impact designs. It is comprised of numerous layer outlines that are associated with a specific example to create an appropriate progression of each of the framework liquid. The outcomes indicated that energy utilization in a solitary impact setup is higher than 13.5 kW, being the energy provided to four modules simultaneously, while in a multi-impact arrangement, energy utilization is under 3.4 kW. Thus, the distillate creation in the single-impact design has an outstanding pattern and turns out to be high at a lower feed temperature. Then again, the distillate creation in the multi-impact setup demonstrated a higher energy utilization although it had an immersion point of 45 °C. In this context, Farzaneh Mahmoudi et al. [8] tried a lab-scale plate-and-casing permeate gap membrane distillation (PGMD) module with 0.12 m^2^ successful film territory. Under various working trial conditions, the feed stream rate was ((0.1–1.1) L/min), temperature input module was (TCi = 15 °C, TEi = 82 °C), penetrate motion differed from (2–12) kg/(m^2^·h), explicit nuclear power utilization was between (1000–2500) kWh/m^3^, and GOR < 1. The exploratory outcomes indicated that when the saline feed stream rate increased at the level of (0.4–1) lit/min, the new water transition increased from 3 to 11 kg/(m^2^·h), and similarly, the nuclear power increased by almost 20%.

For their part, Wafa Suwaileha et al. [9], incorporated a manure-driven forward assimilation inexhaustible fueled film refining framework for bitter water desalination. This framework was used to deliver water for fertigation, recover the weakened draw arrangement, and concentrate consumable water. The key trial results uncovered a water saturation of about 5.7 LMH and a high dismissal of 99.55% at a temperature of 60 °C. Additionally, ideal explicit energy of about 0.32 kWh/m^3^ was accomplished at the most minimal distribution pace of 50 mL/min. Then again, with the use of a concentrated feed arrangement, the MD layer shows less fouling and irrelevant wetting. 

As for Atia E. Khalifa et al. [10], they tentatively contemplated and looked at the presentation of water hole layer refining (MS-WGMD) to multi-stage (MS-AGMD) film refining frameworks for various working conditions. The primary test results uncovered that the yield motion for equal stage-stream associations was higher than that of the arrangement associations. The MS-WGMD framework is discovered to be less delicate to changes in the feed temperature variable and the hole width than the MS-AGMD framework. The equal associations are less delicate to the change in both cold and hot stream rates than the arrangement of stage associations. Subsequently, the particular electric energy utilization is from 5 to 10 kWh/m^3^. Then again, Lan Cheng et al. [11] have tentatively examined and assessed the impacts of the feed temperature, the coolant temperature, the stream rate, the hole warmth conductivity, and the hole width on the motion and acquired yield proportion (GOR) on the air hole film refining (AGMD) and the saturated hole layer refining (PGMD) through self-created empty fiber layer modules with inner energy recuperation. The central trial results uncovered that the transition and GOR in both the AGMD and PGMD were influenced by the hole between the layer and the gathering surface. Accordingly, the improvement of the hole warmth conductivity by a metal net was not proficient to improve the motion and GOR. In the 0.5 mm hole, the motion and the GOR expanded by 7.9% and 59.82% in the PGMD contrasted with the AGMD. Besides, salt maintenance was more prominent than 99.8% in all tests. 

For their part, Aism et al. [12] introduced a close planetary system for a solitary family manor in Dubai. The pilot framework was intended to satisfy the need of 250 L/d. high-temperature water and 15–25 L/d of unadulterated drinking water. The framework utilized the air hole layer refining desalination cycle and level plate gatherer of the opening area of 11.85 m^2^. In this unique situation, the test indicated that this framework was adequate for home-grown boiling water for a solitary family manor of 4–5 people, which can deliver 16 L/d of unadulterated drinking water and 273 L/d of home-grown heated water at a normal temperature of about 50 °C. 

At this point, Guillen-Burrieza et al. [13] have tentatively considered the presentation of a hole film refining sun-powered desalination pilot for a European venture, the MEDESOL project situated in the Solar Platform of Almería (PSA), Spain. The outcomes indicated that the exhibition of the framework is influenced by the temperature of the feed and the stream pace of the liquid stream. The results demonstrated that the presentation proportion of the framework was under 1 and diminished somewhere in the range between 8 and 16% by expanding the saltiness of food from 1 to 35 g/L. It was additionally seen that three multi-stage module frameworks help limit nuclear power utilization. 

As for Winter et al. [14] from the Fraunhofer Institute for Solar Energy, they contemplated frameworks on a winding injury MD-modules with a layer surface region of 5–14 m^2^. The trial results indicated a significant impact of the feed stream and the feed water saltiness on the boundaries and execution of the twisting injury module idea. Besides, during all examinations, the distillate conductivity did not surpass dist = 3.5 S/cm, which is viewed as extremely low. In a starter exploration utilizing a two-venture measure design, it is conceivable to create a high immaculateness distillate with conductivity as low as 0.19 S/cm. 

In their examination, Manna et al. [15] explored another level sheet cross-stream layer module furnished with hydrophobic polyvinylidene fluoride (PVDF) microfiltration film for the expulsion of arsenic from defiled groundwater. At a feed temperature of 60 °C, the acquired yield proportion was discovered to be 0.9, the warm recuperation proportion 0.4, and the water transition 90 kg/(m^2^·h), and an arsenic convergence of 396 ppb, a pervade stream pace of 150 kg/h, and an influent stream pace of 120 kg/h was resolved. A reduction in distillate was seen with expanding arsenic content in the feed water. 

For their part, Raluy, R.G et al. [16] designed and introduced at the Instituto Tecnológico de Canarias (ITC) in Playa de Pozo Izquierdo a sun powered minimal MD show plant. Results were discovered following five years of execution to be palatable. The distillate of the unit differs varies between 5–120 L/d with a conductivity scope of 20–200 μS/cm and explicit nuclear power utilization in the scope of 140–350 kWh/m^3^. 

At this point, Chang et al. [17] examined a sun-based film refining desalination framework SMDDS which uses the air hole type layer refining. The framework is made out of a chilly liquid indoor regulator, hot warm stockpiling tank, siphons, and energy recuperation unit. The outcomes indicated that in radiant days, the distillate was discovered to be 0.258 kg/d while in shady days, the distillate was discovered to be 0.142 kg/d. 

From past research, the hypothetical and exploratory investigations on the penetrate hole layer refining setup are extremely restricted. The point of this work is to consider and assess the exhibition of another model of sun-based desalination with a PGMD standard under various variables (working boundaries, climate conditions).

## 3. Material and Methods

The pilot plant was introduced by the Higher Institute of Applied Science and Technology and was worked under the weather conditions of Kairouan, Tunisia. The distinctive working boundaries (stream rate, water bay, and outlet temperature of every segment of the plant, sun-based radiation) and their effect on efficiency were examined utilizing the control and observing instruments introduced on the plant. Besides, a warm investigation was carried to decide the acquired yield proportion (GOR), the exhibition proportion (PR), and the specific thermal energy consumption (STEC).

### 3.1. Experimental Setup

**Figure 1:**
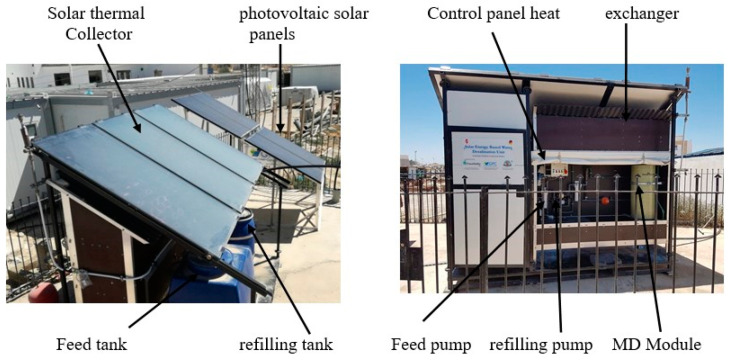
Side and rear views of the experimental setup.

**Figure 2:**
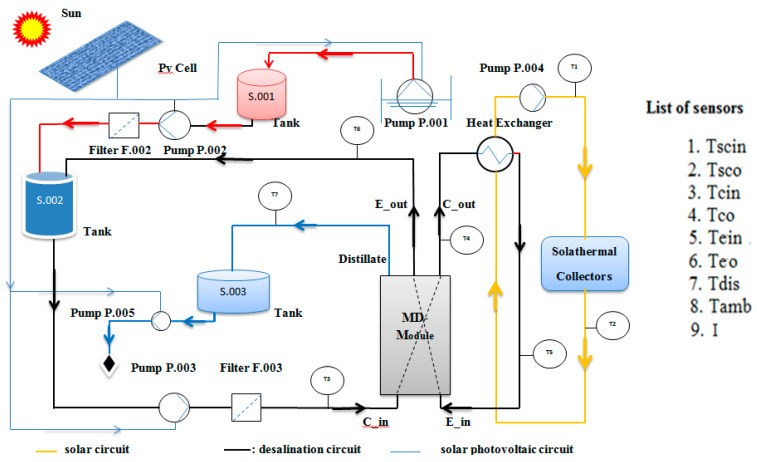
Hydraulic layout of the experimental setup.

**Figure 3:**
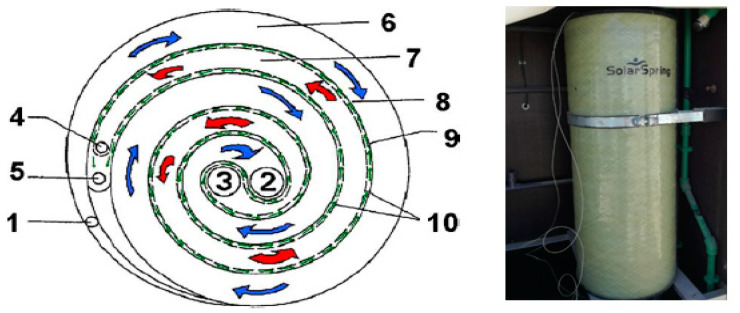
Schematic of the concept of spiral wound module (1) condenser entrance, (2) condenser exit, (3) evaporator entrance, (4) evaporator exit, (5) distillation exit, (6) condenser path, (7) evaporation path, (8) condenser foil, (9) distillation path, and (10) hydrophobic membrane.

Figure 1 shows a photograph diagram of the side and rear views of the experimental setup, while Figure 2 illustrates the hydraulic structure of the experimental setup. Accordingly, the overall system consisted of three interacting circuits. Firstly, the solar circuit provides the thermal energy required to heat the flow. This consists of the solar collector array, the output of which is directed to a heat exchanger that transfers the solar heat from the solar circuit to the MD module [M]. The working liquid in the sun-powered circuit is characteristic mineral water with pH = 7.7 to shield the sun-based gatherers from openness to the pungent feed water. The programmed deaerator is utilized on the sun-powered gatherers to consequently empty the air contained in the liquid during the filling and beginning stages. Furthermore, the desalination circuit comprises the MD module [M], which is a spiral-wound design that provides an effective internal heat recovery [14], as well as a compact arrangement. Then again, the inner warmth recuperation is the warmth recuperated from the cool feed water in the condenser pipe. The construction and format of a winding injury layer with PGMD, which appears in Figure 3, comprises three channels; a condenser channel (Cin and Cout), an evaporator channel (Ein and Eout), and a distillate channel (Dout), and 5 associations. Finally, the condenser and evaporator channels are isolated by the hydrophobic layer. Third, the sun-oriented photovoltaic circle creates the vital electrical energy for the activity of the unit (siphons, PC). The field of photovoltaic authorities consists of 4 modules, DC voltage controllers 12/24 VDC 30, and force inverter 230 V/1 kW. Then, two electrical sun-oriented batteries 12 V/230 AH are utilized to balance out and control the force from the photovoltaic board. The four PV boards are collected in equal and in arrangement with a creating limit of 1 kW. Specialized insights concerning the sun-based gatherer field and other significant parts can be found in Table 1.

### 3.2. Instrumentation

The pilot was outfitted with the fundamental instrumentation for the estimation and control of the different working boundaries and climate conditions. A pyranometer type LP LYPRA 03AC was introduced on a level plane nearby the sun-based authority to gauge the all-out sun-based radiation. Thermocouple type J was introduced to gauge the power source and the channel water temperatures in every unit of the parts and surrounding temperature. At that point, the thermocouple, which gauges the surrounding temperature, is kept in a sanctuary to shield the sensor from direct daylight. Additionally, all the thermocouples are associated with an information securing framework (type Agilent 34970A) to screen and record the obtained signals. Moreover, the Agilent 34970A is associated with the USB port of the PC utilizing a USB link type AB to record varieties in bay and outlet water temperatures during the working time frame. The definite specialized information of the sensors and tests utilized in the exploratory arrangement are shown in Table 2.

## 4. Experimental Results and Discussion

**Figure 4:**
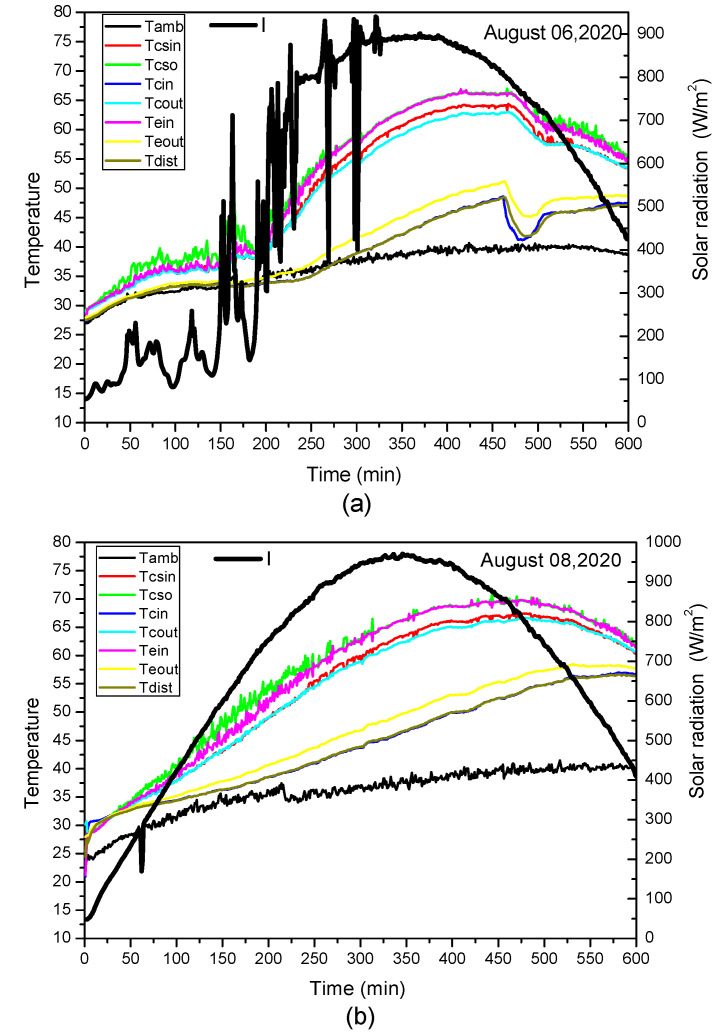

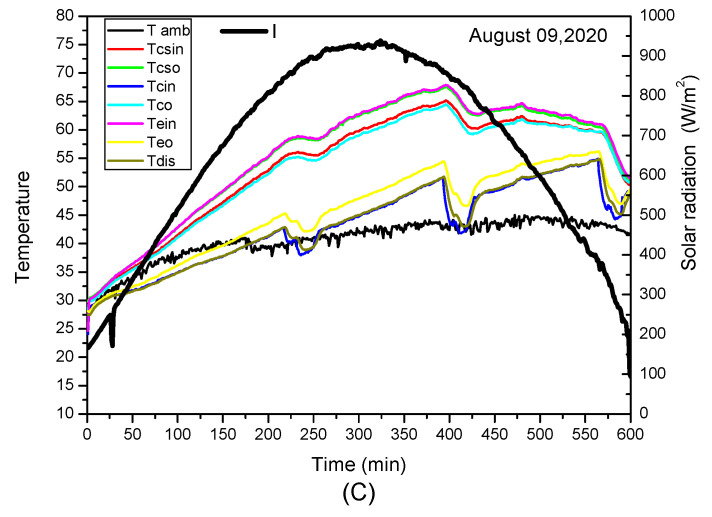
Daily variation inlet/outlet water temperature and solar radiation for different days in August: (**a**) 6 August 2020, (**b**) 8 August 2020, (**c**) 9 August 2020.

The exploratory investigation of the unit was examined during the sunshine hours on various long stretches of August 2020, under the variable sun-based radiation. The trial began at 7:00 am and all boundaries were estimated and recorded every moment until 17:00. Distinctive feed stream rates (200, 300, 400, 500, 600 L/h) were utilized to report the impact of the feed stream rate on creation. Figure 4 shows the everyday varieties of the inlet and outlet water temperature with sun-powered radiation for different long stretches of August. The two days, 8–9 August 2020, were described as a bright day, conversely, 6 August 2020 was viewed as an overcast day. It was recognized that the power source and delta liquid temperature followed patterns similar to the sun-based radiation. It was obvious from the figures underneath that the most noteworthy estimation of sun-based radiation was about 951.37 W/m^2^, which happened around early afternoon on 8 August 2020, while the most elevated encompassing temperature was reached at 13 h 30 m on 9 August 2020 at about 46.13 °C. We noticed that the channel and the power source water temperature of the sun-based authorities followed the conduct of the sun-based radiation. Then again, the most extreme estimations of water outlet enrolled temperatures were 89.86 °C in the late morning. These outcomes demonstrated that the expansion of the warm presentation for the framework was larger than that of the warm exhibition of the sun-oriented authority. The fact is that the sun-oriented authority liquid temperature greatly affects the warm exhibition of the sunlight-based gatherer. 

Table 3 gives data about the normal qualities and the most extreme day-by-day sun-powered radiation, surrounding temperature, outlet water temperature of the sunlight-based authority, delta water temperature evaporator divert notwithstanding the distillate acquired during the activity. At that point, the most extreme creation of 159.20 kg/day was recorded on 9 August at an evaporator delta temperature of 71.91 °C.

### 4.1. The Effect of Global Radiation on the Production Unit

**Figure 5:**
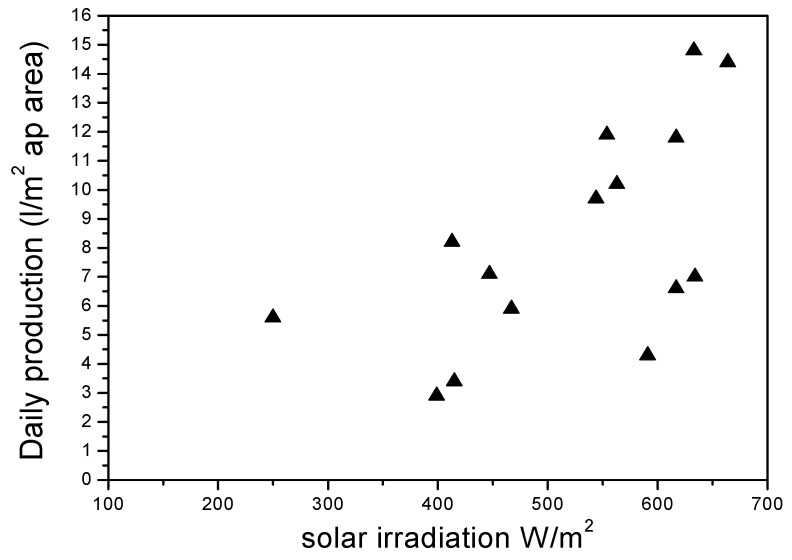
Regular output of fresh water versus solar radiation.

Figure 5 shows day-by-day water profitability as an element of sun-oriented radiation. These outcomes show that there is an immediate impact of sun-based radiation on the measure of the delivered distillate. This shows the creation of the plant increments with the increment of sunlight-based radiation and the other way around for 8 h of persistent activity. The specific purpose behind the observed impact of sunlight-based radiation on new water creation was that the temperature of the feedwater leaving the level plate gatherers increased with the increment in the force of sun-powered radiation. Subsequently, as the feed water temperature at the dissipation channel expanded because of the warming of the sun-based authority, the temperature contrasts across the expanded layer, which prompted an increment in the new water creation of the framework. Moreover, with a sun-oriented gatherer region of 6 m^2^ and no warmth stockpiling tank, the everyday creation came to about 15.92 L/m^2^ assortment territory when the sun-based radiation arrived at its most extreme estimation of 978.27 W/m^2^. The measure of new water accomplished was a promising incentive for an underlying test of the framework. The new water creation of the ebb and flow model was additionally improved by incorporating a control calculation dependent on climate boundaries and a sun-based warmth stockpiling tank into the framework to broaden the activity past nightfall.

### 4.2. Impact of the Feed Stream Rate on Fresh Water Production

**Figure 6:**
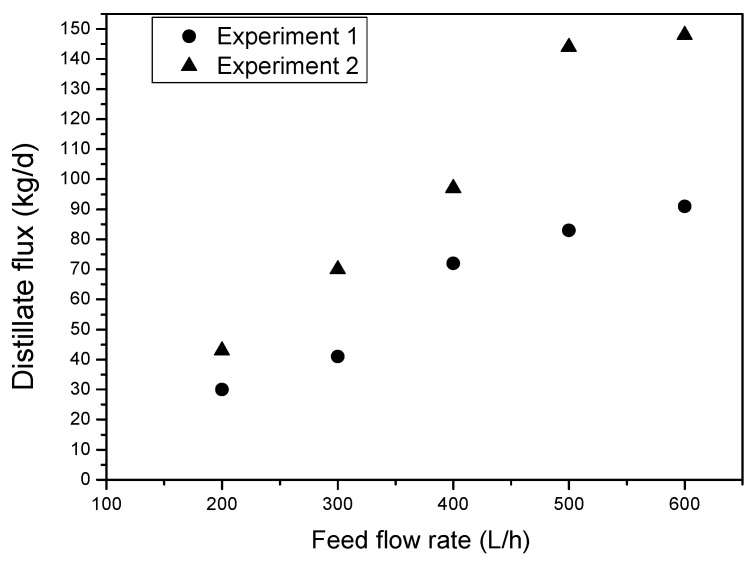
The effect of the feed flow rate on the prototype’s output of freshwater.

Figure 6 shows the impact of the feed water volume stream on the new water creation of the model. We conducted two investigations on various days in August 2020 in which we changed the feed water volume stream from 200 L/h to 600 L/h for five distinct days. The primary perception that we can make from the acquired outcomes is that the creation of the distillate stream expands with the expansion of the feed water volume stream for the two tests. In fact, by raising the feed stream rate from 200 to 600 L/h, the distillate transition increments by 43.33% and 62.63% for analyses 1 and 2, separately. This finding can be attributed to the fact that with the increment in the feed stream rate, the choppiness in the stream channel increments. This builds the quantity of Reynolds, which improves the warmth and mass exchange coefficients in the stream channels and diminishes the impact of temperature polarization at different sides of the layer zone, enhancing the main thrust temperature contrast, and finally, leading to a higher distillate motion. This trial finding was in concurrence with that of Zaragoza et al. [18], who confirmed that the greatest distillate transition was obtained at the most extreme feed stream rate because of a higher disturbance at the higher feed stream rate and lower polarization impact of temperature. The subsequent perception was that the distillate motion acquired during the subsequent test was more noteworthy than the first because of the sun-powered radiation impact. Figure 6 also shows the contrast between the distillate motion estimations of the two tests increments with the increment in the feed stream rate. This may demonstrate that the distillate motion was more sensitive to the stream rate than to the power of the sun-based radiation.

**Figure 7:**
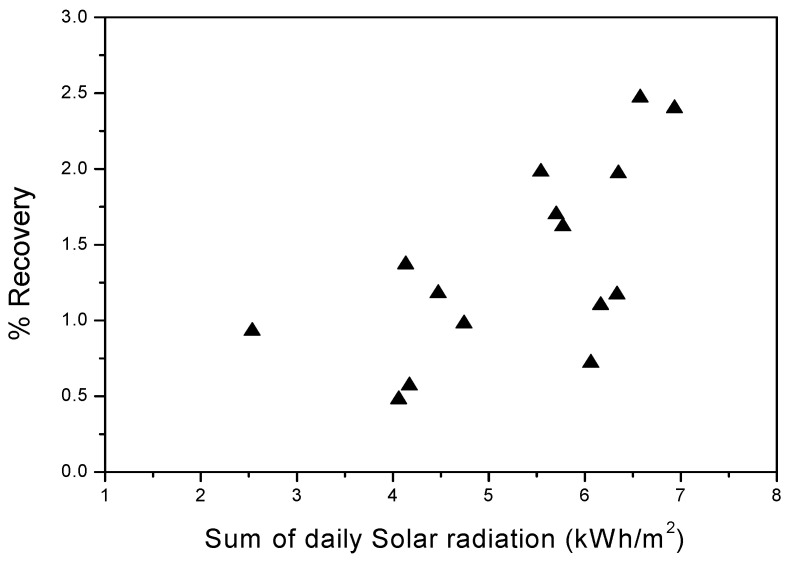
Distillate recovery as a function of solar radiation.

At the point when bitter water vanishes and the fume goes through the pore of the layer, the distillate is framed in the distillate direct and afterward gathers in the distillate tank [S.003], while the rest of the saline solution goes back to the feed tank [S.002]. In fact, along these lines, a portion of the feed is retrieved as distillate. Figure 7 shows the distillate recuperation as a component of sun-powered radiation. Then again, the rate recuperation is characterized by the proportion of distillate stream rate to be considered. This implies that the recuperation rate increments with the increment of the sunlight-based irradiance, which was not yet over 3%. This rate recuperation was in concurrence with the after-effects of past examinations utilizing the winding injury air hole film refining module [19]. An experimental work developed by Banat et al. [20] on a similar membrane distillation system using a direct contact module, showed that the percentage recovery increased considerably with increasing the global irradiation, but it was no more than 4%. One can conclude that direct contact membrane distillation (DCMD) has a greater recovery ratio than PGMD.

## 5. Execution Pointers

Execution markers in warm refining measures are all around characterized, for the most part, unaltered across refining advancements and omnipresent in test works, among which the accompanying were chosen for this examination: 

### 5.1. Explicit Thermal Power Utilization or STEC

The warmth energy is needed to deliver a unit volume of penetrate. This presentation pointer is basic among all warm refining advancements and perhaps the most broadly utilized benchmark of warm execution. 

The particular nuclear power utilization is considered as the proportion of the energy the warmth exchanger supplies to the amount of the distillate produced.

(1) $$\text{STEC}=\frac{Q_{HX}}{{\dot{m}}_{p}}$$

The STEC can be extended and modified by the accompanying basic thermodynamic condition.

(2) $$\text{STEC}=\frac{{\dot{m}}_{f}C_{pf}(T_{eout}-T_{ein})}{{\dot{m}}_{p}}$$

**Figure 8:**
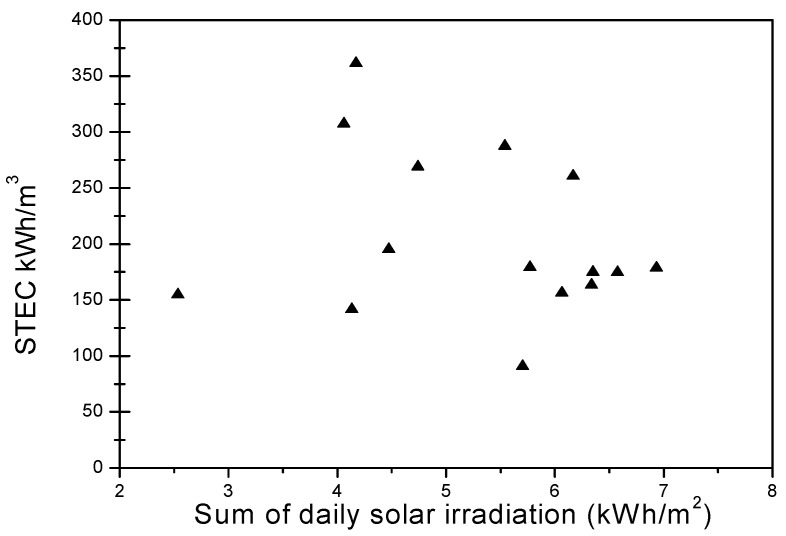
Influence on the STEC of regular daily radiation.

From Figure 8, it can be observed that the particular warmth energy utilization determined for this framework is in the scope of 90 to 310 kWh/m^3^. Nonetheless, this outcome is frail when contrasted with other desalination measures, for example, sunlight-based still (640 kWh/m^3^) [20]. Hence, the MD cycle could be vigorously intensified with other desalination advances.

### 5.2. Gained Output Ratio or GOR

GOR is a worldwide proportion of the energy proficiency of the framework. A higher acquired yield proportion relates to a lower energy utilization for every unit of the delivered distillate. GOR is a more itemized pointer of energy proficiency measure. Then, being very similar to the STEC, the GOR can be considered as the proportion of the warmth needed to disintegrate the saturate and the real provided heat. Moreover, it is generally utilized as an exhibition marker, and hence, can be presented as follows:

(3) $$\text{GOR}=\frac{{\dot{m}}_{p}\Delta H_{V}}{{\dot{m}}_{f}C_{pf}\left(T_{eout}-T_{ein}\right)}$$

**Figure 9:**
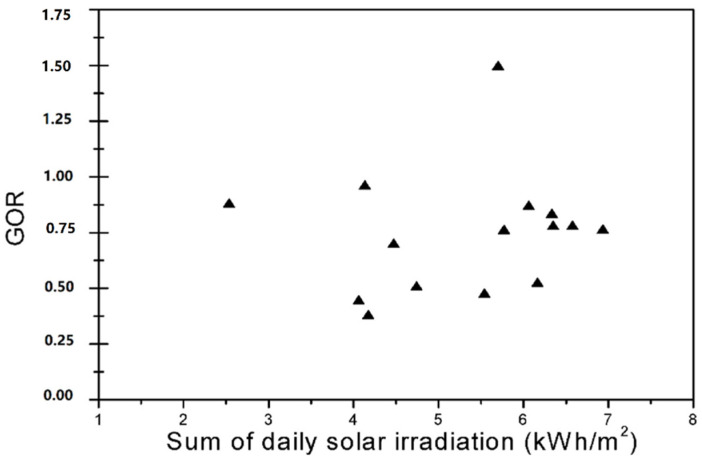
The effect of regular radiation on the output ratio gained.

As can be seen in Figure 9, contingent upon the day-by-day sun-oriented radiation, the GOR after-effects of this framework are in the scope of 0.3 and 1.4. Banat et al. [20] calculated the GOR of a DCMD module at mid-noon of each day and found that the GOR varied from one day to another depending on the sum of daily irradiation and was in the range of 0.3–0.9. Thus, the current system presents a greater GOR.

## 6. Distillate Quality

**Figure 10:**
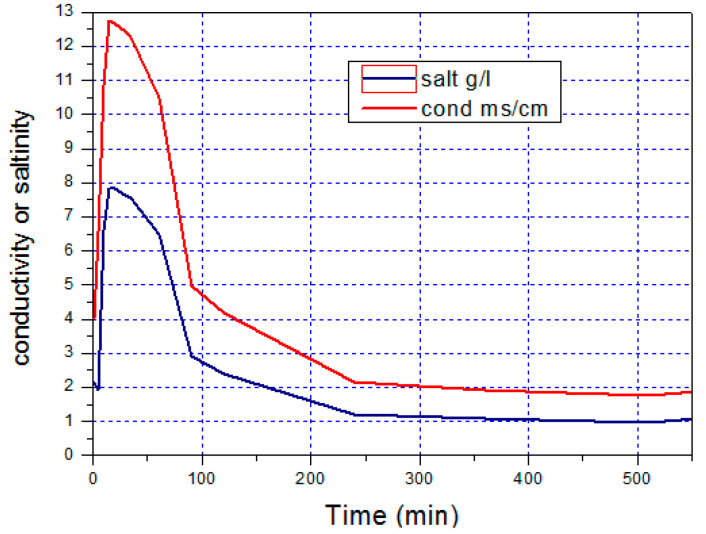
Variation of conductivity and salinity.

In light of a few led tests, numerous scientists indicated that the dismissal pace of MD is assumed to be high. In this way, the estimations of the distillate conductivity are performed to check the usefulness of the twisting loop module and assess the nature of the distillate. Moreover, since the pressing factor level on the evaporator and condenser channels is more noteworthy than that of the distillate channels, it means that any blemish or deformity would bring about an expansion of conductivity, for example, the expansion of the salt substance of the distillate. The feed water with a conductivity of k feed = 18,480 μS/cm is roughly 11.75 g/L and utilized with a volume stream pace of 400 kg/h. The consequences of the distillate water conductivity acquired over the long haul are presented in Figure 10.

Over a few minutes, the deliberate conductivity is viewed as high and enlisted at about 12,750 μS/cm, which is around 7.82 g/L, and drops afterward. After 2 h of activity, the conductivity reached an estimation of k_dist_ = 4180 μS/cm, which was roughly 2.4 g/L, at that point then, after 5 h of activity, the worth gets steady at about k_dist_ = 1865 μS/cm, which was around 1.044 g/L of saltiness. 

## 7. Economic Study

The determination of the cost of freshwater production and the payback period of the PGMD process is based on a variety of parameters. In general, the investigation method depends on the calculation of capital and operating costs. Therefore, the total once-a-year cost of desalination, *C_tot_*, is determined as the sum of the annual amortization or fixed cost, *C_fixed_*, and the annual maintenance and operating cost, *C_o&m_*:

(4) $$C_{tot}=aC_{fixed}+C_{o\&m}$$

The payments over time (amortization) factor, *a*, is a function of the year or lifetime of the unit, *n*, and the annual interest rate (%), *i*:

(5) $$a=\frac{i{\left(1+i\right)}^{n}}{{\left(1+i\right)}^{n}-1}$$

The unit cost of produced water, *WPC*, is the ratio of the total annual cost to the quantity produced annually by the prototype:

(6) $$WPC=\frac{C_{tot}}{f{\dot{m}}_{dis}365}$$

Since the solar membrane distillation plant is not in quotidian operation, a plant availability factor (*f*) was introduced to adjust the annual expenditure. The cost analysis was based on a plant availability factor of 90%, a unit life of 20 years, and an interest rate of 5%. The land was offered by the University of Kairouan, Tunisia. Table 4 and Table 5 show, respectively, the cost of each purchased component constituting the experimental prototype and a summary of the results of the cost evaluation of the experimental prototype.

## 8. Conclusions

The trial investigation of the pilot solar-powered saturate hole membrane distillation was observed for various days in August 2020 under the real climate of Kairouan, Tunisia. The obtained results show that the sun-oriented radiation and feed water decidedly influence the pervade creation of the MD module where the everyday creation reaches about 15.92 L/m^2^ ap with an estimated electrical conductivity of the distillate of 1865 μS/cm and 1.044 g/L saltiness. The particular nuclear power utilization determined for this framework goes from 90 to 310 kWh/m^3^. The GOR of the layer module shifted starting with one day, then onto the next, relying upon the day-by-day sunlight-based radiation. The GOR after-effects of this framework were in the scope of 0.3–1.4.

## Figures and Tables

**Table 1:** Technical details of the solar collector field and other important components.

Water Solar Collector		Membrane Distillation		Pv Monocrystalline Module	
Area	2 m^2^	Area	10 m^2^	Temperature range	−40 to 85 °C
Tube material	copper	Module-height	gross = 900 mm net = 725 mm	Module dimension	1.640 × 992 mm
Tube thickness	0.002 m	Material	Polytetrafluoroethylene (PTFE)	weight	21 Kg
Rise-outer diameter	0.0127 m	Length	73 mm	Impp	8.07 A
Rise thickness	0.56 × 10	Pore diameter	0.1–0.4 μm	Vmpp	30.55 V
Thickness of insulation	0.1 m	nature separation	hydrophobic	Wp	245 W
Weight	48 kg	condenser diameter	20 mm		
Angle	45°	Evaporator diameter	20 mm		
Loss coefficient	4.8 W/m^2^ k	distillate diameter	20 mm		

**Table 2:** Technical specification of sensors and probes used in the experimental set up.

Component	Type	Description	Value
Thermocouple	Type J	Temperature sensors	−50 °C–400 °C
Pyranometer	LP LYPRA 03AC	Measuring Range	0–2000 W/m^2^
Pump	Shurflo LS2255	Refilling	Open flow 13.6 L/mn
Pump	Shurflo LS2255	Feed	Open flow 13.6 L/mn
Feed volume flow	60/185(2/96)	H_2_O with 20 °C	--
		iden Nr: 15002	
PLC	Agilent BenchLink	Data Logger 3 Agilent 34970a	--
Conductivity Meter	CD-4307SD	Measuring cd, salt	0 to 200 ms, 0 to 12% salt (% weight)

**Table 3:** Results of some measures of the average values and daily maximum for various days.

Parameter	Unit	2 August 2020	3 August 2020	6 August 2020	8 August 2020	9 August 2020
Avg. Solar Radiation	W/m^2^	601.3	637.62	584.61	682.22	677.56
Max. solar radiation	W/m^2^	859.6	896.7235	939.98	951.37	978.27
m_feed_	kg/h	200	300	400	500	600
distillat	kg/d	46.60	73.40	101.80	142.60	159.20
Avg. T_amb_	°C	40.908	42.23	38.39	39.87	42.61
Max. T_amb_	°C	44.773	47.99	40.64	41.47	46.13
Avg. T_sco_	°C	59.53	65.44	54.41	58.13	57.37
Max. T_sco_	°C	88.21	89.86	66.99	69.79	71.58
Avg. T_ein_	°C	52.73	57.25	53.51	57.39	59.71
Max. T_ein_	°C	76.08	71.884	66.88	70.211	71.91

**Table 4:** Investment cost of each component constituting the experimental prototype.

Prototype Components	Quantity	Cost (€)	Total Cost (€)
Photovoltaic module 245 W	4	188.5	754
Thermal solar collector 2 m^2^	3	214.6	643.8
Compact distillation module (PGMD, Pumps, filters, feed tank, and heat exchanger)	1	11,000	11,000
Piping/Connection accessory	1	681.5	681.5
Circulation pump	1	101.5	101.5
DC pumping system	1	1044	1044
DC/AC protection box	1	275.5	275.5
Wiring and cable routing	1	217.5	217.5
Battery/charge regulator/Inverter	1	788.8	788.8
Structure	1	174	174

**Table 5:** Summary of the cost evaluation results of the experimental prototype.

Capital cost	15,680.6 €
Operating and maintenance costs (*C_o&m_*)	250,886 € year^−1^
Lifetime (*n*)	20 year
Interest rate (*i*)	5%
Amortization factor (*a*)	0.080 year^−1^
Fixed charges (*C_fixed_*)	125,444 € year^−1^
Total unit cost	150,533 € year^−1^
Unit availability (*f*)	90%
Unit capacity	0.148 m^3^ d^−1^
Water production cost (*WPC*)	30.96 € m^−3^
Net earning	30.95
Payback period (*Pb*)	506.60 d

## Data Availability

Not applicable.

## Nomenclature

I	sunlight based radiation (W/m^2^)
T_amb_	surrounding temperature (°C)
T_scin_	channel water temperature of the water sunlight based authority (°C)
T_sco_	outlet water temperature of the water sunlight based gatherer (°C)
T_ein_	entrance water temperature of the channel of the evaporator (°C)
T_eout_	outlet water temperature of the channel of the evaporator (°C)
T_cin_	entrance water temperature of the condenser channel (°C)
T_cout_	outlet water temperature of the condenser channel (°C)
T_dist_	water temperature of the distillate (°C)
Q_HX_	complete energy input needed for the refining cycle [kWh]
m_P_	mass stream pace of pervade creation [kg/h]
m_F_	mass stream pace of the feed water [kg/h]
ΔH_v_	water idle warmth of vaporization in [kj/kg]
A_solar_	absolute opening zone of the sun based authority field [m^2^]
m_solar_	mass stream rate in the sunlight based gatherer field [kg/s]
C_PF_	isobaric explicit warmth limit of the feed water [kJ/ (kg °C)]
